# Prevalence of late and long-term effects of cancer (treatment) and use of complementary and alternative medicine in Norway

**DOI:** 10.1186/s12906-022-03790-z

**Published:** 2022-12-05

**Authors:** Agnete E. Kristoffersen, Barbara Wider, Jorunn V. Nilsen, Mona Bjelland, Dana C. Mora, Johanna Hök Nordberg, Ann Ragnhild Broderstad, Kiwumulo Nakandi, Trine Stub

**Affiliations:** 1 National Research Center in Complementary and Alternative Medicine (NAFKAM), Department of Community Medicine, UiT The Arctic University of Norway, Tromsø, Norway; 2 The Norwegian Cancer Society, Oslo, Norway; 3 Regional Cancer Center Stockholm Gotland, Stockholm, Sweden; 4 Karolinska Institutet, Department of Neurobiology, Care Sciences & Society, Division of Nursing & Department of Physiology & Pharmacology, Stockholm, Sweden; 5 Centre for Sami Health Research, Department of Community Medicine, UiT The Arctic University of Norway, Tromsø, Norway

**Keywords:** Cancer, Late effects, Long-term effects, Complementary therapies, Supportive care, CAM

## Abstract

**Background:**

The increasing number of patients surviving cancer leads to more people experiencing late and long term-effects from the disease and its treatment. Fatigue, sleep disorders, early menopause, pain, and nerve damage are commonly reported. Methods helping people to recover after cancer treatment are therefore essential. The aims of this study were threefold; (1) to determine the level of cancer patients suffering from late and long-term effects of cancer diagnosis and treatment in Norway, (2) explore complementary and alternative medicine (CAM) modalities used for managing these adversities, and (3) describe self-perceived benefits and harms of the CAM interventions.

**Methods:**

The study was conducted in cooperation with the Norwegian Cancer Society (NCS) and consisted of an online cross-sectional study among members of the NCS user panel with present or previous cancer (*n* = 706). The study was carried out in September/October 2021 using a modified cancer-specific version of the International Questionnaire to Measure Use of Complementary and Alternative Medicine (I-CAM-Q). A total of 315 women and 153 men agreed to participate, resulting in a response rate of 67%.

**Results:**

Most of the participants (83%) suffered from late and long-term effects of cancer treatment; mostly fatigue (59.2%), sleep disorder (41.5%), hot flashes (39.2%), nerve damage (polyneuropathy, 38.0%), and pain (36.6%) with a mean number of 5.1 different late and long-term effects. Late and long-term effects were positively associated with younger age and college/university education. Nearly half of the participants experiencing late and long-term effects (43%) reported having used CAM to treat these complaints. Most frequently used were self-help practices (26%) such as relaxation therapy (19%), yoga (14%) and meditation (13%), but also visits to CAM providers were reported by 22%. Herbal- and other natural remedies to treat late and long-term effects were used by 13%. A high percentage of CAM users reported self-perceived improvements of their symptoms (86% for self-help practices, 90% for visits to CAM providers). Few experienced adverse effects of the CAM treatment.

**Conclusion:**

A large proportion of cancer patients suffered from a wide range of late and long-term effects of cancer diagnosis and treatment, and they use CAM to treat these complaints to a rather high degree. Relaxation therapy, yoga, meditation, massage, and acupuncture were the most frequently used therapies regardless of complaint. The therapies used are generally considered to be both safe and beneficial for the respective complaint, indicating that the participants seem to be well informed about the choices they make.

## Background

A total of 36,998 new cases of cancer were reported in Norway in 2021, with prostate (14%, *n* = 5188), breast (10%, *n* = 4023), lung (10%, *n* = 3499), and colon cancer (9%, *n* = 3204) as the most frequent cancer types [1]. The number of cancer survivors has been increasing and by the end of 2021, there were 316,145 people who had previously been diagnosed with cancer living in Norway [1]. Due to early detection, increasing number of treatment options and lines, and more targeted treatment methods, nearly three out of four patients survive cancer for 5 years or longer and they live longer with their disease [2].

The increasing number of patients surviving cancer leads to more people experiencing late and long-term effects of cancer diagnosis and treatment [3]. Late and long-term effects are understood as an adverse effect or complication of the cancer or its treatment that lasts for more than 1 year after the end of treatment, or an adverse effect or a health condition likely to be due to the cancer diagnosis or treatment, and which occurs 1 year or more after the end of treatment [4]. Some may develop during treatment and persist *(long-term effects*) such as fatigue, whereas others may develop many years later (*late effects)* such as secondary cancer or cardiovascular diseases [5]. The more intensive and invasive the treatment is, the greater the risk of having to live with late and long-term effects of the treatment [6]. Surgery, chemotherapy, and radiation have all potential negative effects. This includes physical (e.g., secondary cancers, cardiopulmonary problems, fatigue, neuropathy, oral problems, musculoskeletal disorders, and lymphedema), psychosocial (e.g., anxiety and depression), and cognitive difficulties (e.g., concentration, loss of memory and dementia) [7–9]. Significant increases in morbidity associated with treatment-related complications have been found up to 25 years after the initial diagnosis. A Norwegian study from 2017 [10] reported that up to 35% of cancer survivors experience chronic fatigue. Regardless of when they occur, late and long-term effects can significantly impair physical, psychological, or social functioning, and thus reduce cancer survivors’ quality of life [11–14].

In Norway, almost 50% of the people with cancer are of working age [15] but less likely to be employed after cancer treatment than the general population [16], although an average of 67% of cancer patients return to work after cancer. A lower proportion among women, those without university education and those who have heavy physical work as well as those with older age return to work. Cancer patients are also shown to have a loss of income that averages 10–15% [4]. Annual income and work abilities are particularly low for central nervous system tumor survivors [17], and for patients treated with radiation therapy [18]. A qualitative study of colorectal cancer patients’ pathways in Norway showed that various late and long-term effects had unique impact also on people’s everyday life: A mother suffered because she lacked the energy to fulfill her role as a care-giver and a nature lover lacked the energy to spend time outdoors because of a radiation injury [19]. Hence, late and long-term effects from cancer diagnosis and treatment have large socioeconomic as well as personal consequences. When the cancer disease is associated with other significant impairments such as late and long-term effects, the use of complementary and alternative medicine (CAM) is expected to be high [20]. CAM is the term used for medicinal products and practices that are not part of standard medical care [15], and that are mainly offered outside public health care [21].

*Cancer-related fatigue* (CRF) is one of the most frequently reported late and long-term effects of cancer diagnosis and treatment [22]. Up to 35% of those who have completed curative treatment after for example lymphoma, breast, cervical and testicular cancer, and without known residual disease, will experience chronic fatigue after completion of treatment [4, 10, 23, 24] in comparison to 11% in the general Norwegian population [25]. CRF varies in severity and leads to weakness, lack of energy, and decreased mental capacity and cognition [26], and interferes significantly with usual functioning due to physical, emotional, and cognitive exhaustion [27]. The general approach to CRF management includes education and counseling, physical activity, psychosocial interventions, and limited pharmacological options are available [4, 28]. As conventional therapies for cancer-related fatigue management are suboptimal, many of those suffering from fatigue use CAM [29].

*Sleep disturbances* are estimated to occur in 35–75% of patients with cancer [30] compared to in 21% of the general population across Europe [31]. Sleep difficulties in cancer patients can be caused by pain, restlessness and worries [32]. Depending on the underlying cause(s) of sleep disturbances, conventional treatment is based on lifestyle and behavior changes, psychological therapies (e.g., cognitive behavioral therapy), and/or medication. Due to limitations of pharmaceutical and psychological treatments, patients explore CAM modalities as a suitable treatment option [33].

The joint burden of cancer and menopause impacts millions of women globally [34]. *Menopausal estrogen deprivation* causes physiological and psychological symptoms like hot flushes and night sweats. Approximately two out of three breast cancer survivors experience this with strong impact on quality of life [33]. Systemic menopausal hormone therapy provides symptom control and may be used after most cancers but should be avoided after estrogen-dependent cancers. Non-hormonal methods to manage vasomotor symptoms include cognitive behavioral therapy, hypnosis, selective serotonin reuptake inhibitors, serotonin noradrenaline reuptake inhibitors, clonidine, and gabapentin [34]. Despite a rich variation of conventional treatment options, many women turn to CAM for symptom relief [33].

*Chemotherapy-induced peripheral neuropathy (CIPN)* is a frequent adverse effect experienced by cancer patients treated with chemotherapy [35] with a prevalence of 68% within the first month post-chemotherapy, 60% at 3 months [36], and 30-50% at 6 months or later depending on the chemotherapy used [36, 37]. CIPN presents most often as sensory polyneuropathy, manifesting as pain, paresthesia, or a burning sensation [38, 39]. Efficacious pharmacological therapeutic options for patients with established CIPN are limited [40]; currently there is no consistent evidence of efficacy for any drug to prevent these challenging adverse effects [39]. Patients are advised to avoid factors that can aggravate nerve damage like smoking, high alcohol consumption and sitting with legs crossed and are encouraged to be physically active [37]. When patients experience chronic CIPN, treatment approaches focus on reduction or relief of neuropathic pain [40]. Acupuncture trials in patients with CIPN have suggested that acupuncture may alleviate CIPN symptoms and improve nerve conduction [41–44], but data are still limited.

*Cancer-related pain* is one of the major burdens on cancer survivors and has a strong impact on quality of life [45]. A large number of cancer patients in Europe (72%) suffer from cancer-related pain, of whom 56% reported moderate to severe pain on a monthly basis [46]. Analgesic drugs are only one part of cancer pain management and a variety of non-invasive techniques such as psychological and rehabilitative interventions are recommended [47]. Despite this, it often remains underdiagnosed, poorly evaluated, and insufficiently treated within conventional health care [46, 48, 49]. This might be the reason why many cancer survivors look for other treatment options for this complaint. A recent overview of 27 systematic reviews investigating CAM for cancer pain found that psychoeducational interventions; music interventions, acupuncture plus drug therapy; Chinese herbal medicine plus cancer therapy; compound kushen injection; reflexology; lycopene, transcutaneous electrical nerve stimulation (TENS); qigong; cupping; cannabis; Reiki; homeopathy (Traumeel); and creative art therapies might have beneficial effects on adult cancer pain [50].

More than 75% of all cancer patients reported experiencing acute *cognitive symptoms* during chemotherapy and 17–34% of them have long-term post-treatment cognitive deficits lasting up to ten years [51]. This can significantly affect cancer survivors’ quality of life [33] with regard to attention, executive function, speed of information processing, language, psychomotor function, visuospatial skill, verbal and visual memory [52]. Pharmacological agents that have been studied include psychostimulants, erythropoietin, and hormonal (supplement) treatments for patients who receive hormonal suppression therapy. In addition, several cognitive rehabilitation programs have been evaluated. Recently, the approach of physical exercise to treat cognitive deficits has received great interest [53]. Although few studies have explored CAM treatment for chemotherapy-induced impairment, electroacupuncture trigeminal nerve stimulation plus body acupuncture may be beneficial [54]. This could potentially reduce chemotherapy-induced working memory impairment and the incidence of certain digestive, neurological, and distress-related symptoms and serve as an effective intervention for cancer patients under and post chemotherapy treatment.

The prevalence of *lymphedema,* the build-up of fluid in soft body tissues in the arm after treatment for breast cancer has been shown to be around 20% after armpit dissection, and around 5% after sentinel lymph node surgery [55]. The incidence of lymphedema after treatment for gynecological cancer has been reported to be between 0 and 50% [56, 57]. The highest prevalence is found in those who have had lymph node surgery performed in the groin and pelvis [56]. For many patients with lymphedema, lifelong treatment may be needed to avoid exacerbating the condition. Treatment strategies include physical activity, lifestyle changes, circulation exercises [4], elevation, complete decongestive physiotherapy, pneumatic pumps, and, after failure of all other methods, surgery [58]. Patients are also advised to avoid situations that can puncture or damage the skin to prevent infections [59].

To improve lymphedema symptoms, research has also shown that acupuncture [60, 61], moxibustion [61], massage [62], and ayurvedic medicine (yoga specifically) [63], and osteopathic manipulative treatments (OMT) may be beneficial [33, 64]. Although acupuncture appear to be safe and well tolerated [65, 66], needling in the area of the lymphedema should be avoided as lymphedematous skin is at risk for recurrent infections, including cellulitis, erysipelas, and lymphangitis [67, 68]. Cellulitis is a well-described complication of lymphedema, particularly in patients who have undergone axillary or inguinal lymph node dissection [69].

The prevalence of *depression* and *anxiety* following cancer and cancer treatment was found to be 11.6 and 17.9% in a systematic review and meta-analysis among long-term cancer survivors [70], not much higher than the 12-month prevalence of 10 and 15% in the general Norwegian population [71]. As antidepressants can worsen existing cancer symptoms and interact with chemotherapy agents [72], other approaches are needed. Sertraline and citalopram tend to have few interactions and are generally well tolerated as first line agents while cognitive-behavioral therapy can be useful in means of understanding the thoughts, feelings, and behaviors that cause or maintain depression and anxiety [72]. With regard to CAM, studies report positive effects of yoga [73], meditation [74, 75], and massage therapy [76] for anxiety and depression in cancer patients.

Limited knowledge of the connection between previous cancer treatment and late and long-term effects that may occur many years after completion of treatment, has been a challenge. In a 2018 survey conducted by the Norwegian Cancer Society (NCS), only 38% of the cancer patients reported to have received sufficient information about possible late and long-term effects that could occur from received cancer treatment. However, almost half of the respondents (47%) found that health care providers they were in contact with had sufficient knowledge of late and long-term effects [77].

The aims of this study were threefold; (1) to determine the level of cancer patients suffering from late and long-term effects of cancer diagnosis and treatment in Norway, (2) explore CAM modalities used for handling these adversities, and (3) self-perceived benefits and harms of the CAM interventions.

## Methods

In cooperation with the Norwegian Cancer Society (NCS), an online cross-sectional study was conducted among the members of their user panel with present or previous cancer (*n* = 706). The study was carried out in 2021 using a cancer-adjusted version of the International Questionnaire to Measure Use of Complementary and Alternative Medicine (I-CAM-Q) [78].

### Participants

The NCS’s user panel is a web panel of people with experience of cancer either as cancer patients or relatives of cancer patients. The panel consists of 706 people with previous or present cancer, more women (75%) than men (25%). The members are recruited through social media, the NCS’s webpage, and social events. All members of the NCS’s user panel with present or previous cancer aged 18 years or above were invited to participate in the survey.

### Recruitment and data collection

**Fig. 1:**
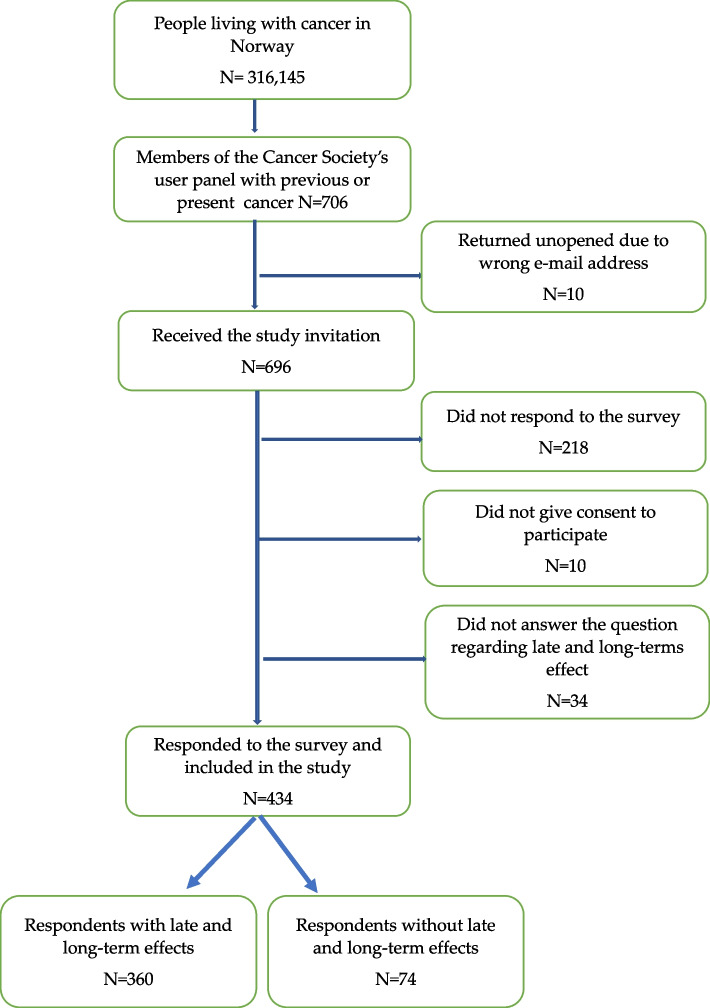
Flow chart of the included participants

All members who fulfilled the inclusion criteria (*n* = 706) received a request from the NCS by e-mail with a link to the online survey. The first page was an information letter where participants had to tick “I agree to participate” in order to continue to the survey. The survey was distributed online only. Due to 10 e-mails returned as undeliverable, the invitation was received by 696 members of the NCS’s user panel. Of the 478 members who responded 468 agreed to participate resulting in a response rate of 67.2% (Fig. 1).

Excluded from the analyses were participants who did not answer the question regarding late and long-term effects (*n* = 34). This led to a study population of 434 participants.

### Measures

A cancer-adapted version of the International Questionnaire to Measure Use of Complementary and Alternative Medicine (I-CAM-Q )[78] was used to collect data on visits to CAM providers, use of natural remedies, and self-help practices to treat late and long-term effects of cancer. For all modalities used, the participants were asked follow-up questions about reason(s) for CAM use where CAM modalities used to *Treat adverse effects / late and long-term effects of the cancer diagnosis and treatment* were included in the analyzes*.* Other options were (1) *To treat/slow down the cancer or prevent the cancer from spreading*; (2) *Strengthen the body / immune system*; (3) *Increase quality of life, coping, relaxation or well-being*; and (4) *Other reasons* which were analyzed and described in a previous paper [79]. Questions regarding late and long-term effects of cancer were collected using the following question: *Have you suffered of late or long-term effects from your cancer diagnosis and treatment?* with the following response options: *No, yes,* and *don’t know*. All who replied *yes* were asked to specify what late or long-term effects they had experienced with the following complaints listed: *fatigue; hot flashes; early menopause; reduced fertility; cognitive challenges; mouth/tooth problems and reduced taste; diarrhea; constipation; unwanted weight loss; unwanted weight gain; urinary tract problems; lymphedema; nerve damage (polyneuropathy); decreased muscle strength and mobility; pain; anxiety or depression; sleep disorders; heart and lung problems; sexual problems;* and *other late and long-term effects.*

### Measures of personal characteristics

Demographic data such as income and education were also collected. Data on age, gender, and cancer diagnosis had already been collected from all members by NCS when they signed up as members of the user panel and were added to the survey responses for all participants.

Age was an open question and assessed as a continuous variable as well as categorical after being merged into the following groups: *19-50 years*, *51-64 years,* and *65-82 years* (the age range of the participants).

Level of education was collected using four categories: 1. *Primary school up to 10 years*; 2. *Secondary school 10-12 years*; 3. *College/university less than 4 years*; and 4. *College/university 4 years or more*.

Household income was collected using the following categories *NOK < 400,000/EUR < 40,000*; *NOK 400,000-799,000/EUR 40,000-79,900*, and *NOK 800,000/EUR 80,000 or more* in addition to an option not to provide income information.

Other personal characteristics included sex (*female*, *male*) and residence (merged into the main Norwegian regions of *South-East*, *South*, *West*, *Central*, and *North*).

#### Statistics/ power calculation

With a margin of error of 5%, a confidence level of 95%, and a heterogeneity of 50%, we needed a minimum sample of *n* = 384 to represent the Norwegian cancer population of 316,145 for adequate study power [80]. Descriptive statistics were carried out using Cross-tabulation and frequency analyses while Pearson chi-square tests and Fisher exact tests were used for between group analyses of categorical variables with binary logistic regression for adjusted values. For continuous variables, independent sample t-tests were used. Significance level were set at *p* < 0.05. The analyses were conducted using SPSS V.28.0 for Windows.

## Results

### Basic characteristics of the participants

The members of the NCS’s user panel consist of more women (75.0%) than men (25.0%) resulting in more women than men in the study (67.1% versus 32.9%, Table 1). The participants had a mean age of 59.2 years (range 19-82 years), and most participants had a high income (46.4%), a college or university education (63.3%), and were living in the South-Eastern part of Norway (53.0%). Most of the participants lived with a spouse/partner (72.1%, Table 1).

**Table 1:** Basic characteristics of the participants and associations for late and long-term effects

	Total		Sex					Late and long-term effects				
			Women		Men		***p***-value	Yes		No / don’t know		***p***-value
	%	***n*** = 434	%	***n*** = 291	%	***n*** = 143		%	***n*** = 360	%	***n*** = 74	
**Sex**												0.127*
Women	67.1	291						68.6	247	59.5	44	
Men	32.9	143						31.4	113	40.5	30	
**Age**							< 0.001*					< 0.001*
19-50 years	23.1	100	27.9	81	13.3	19		23.4	84	21.6	16	
51-64 years	41.3	179	43.4	126	37.1	53		44.8	161	24.3	18	
65-82 years	35.6	154	28.6	83	49.7	71		31.8	114	54.1	40	
Mean age years (SD)	59.18 (11.295)		57.34 (11.277)		62.92 (10.408)		< 0.001**	58.56 (10.594)		62.22 (13.906)		0.011**
**Education**							0.199*					0.038*
Primary school	6.5	28	5.2	15	9.1	13		5.3	19	12.2	9	
Secondary school	30.3	131	29.3	85	32.2	46		29.2	105	35.1	26	
College / University	63.3	247	65.5	190	58.7	84		65.5	235	52.7	36	
**Household income**							0.477*					0.545*
Low (Less than EUR 40,000)	10.4	45	10.3	30	10.5	15		9.7	35	13.5	10	
Middle (EUR 40,000 to 79,000)	35.1	152	35.9	104	33.6	48		35.9	129	31.1	23	
High (EUR 80,000 or more)	46.4	201	44.5	129	50.3	72		46.8	168	44.6	33	
No reply	8.1	35	9.3	27	5.6	8		7.5	27	10.8	8	
**Household**^**1**^												
Live alone	22.4	97	24.7	72	17.5	25	0.088*	23.1	83	18.9	14	0.437*
Live with a partner	72.1	313	68.0	198	80.4	115	0.007*	71.9	259	73.0	54	0.857*
Live with own children	19.6	85	23.0	67	12.6	18	0.010*	19.7	71	18.9	14	0.874*
Other	1.6	7	1.7	5	1.4	2	0.580^	1.1	4	4.1	3	0.067*
**Place of residence (region)**							0.456*					0.210^
South-East	53.0	230	54.3	158	50.3	72		51.9	187	58.1	43	
South	4.6	20	4.5	13	4.9	7		4.2	15	6.8	5	
West	24.2	105	22.0	64	28.7	41		23.9	86	25.7	19	
Central (Trøndelag)	7.8	34	7.6	22	8.4	12		8.3	30	5.4	4	
North	10.4	45	11.7	34	7.7	11		11.7	42	4.1	3	
**Cancer site**^**1**^												
Breast	38.7	168	57.4	167	0.7	1	< 0.001*	40.6	146	29.7	22	0.082*
Gastrointestinal	14.3	62	10.7	31	21.7	31	0.002*	13.3	48	18.9	14	0.211*
Male genitalia	11.1	48	0.0	–	33.6	48	–	10.0	36	16.2	12	0.120*
Female genitalia	8.3	36	12.4	36	–	–	–	9.4	34	2.7	2	0.055*
Lymphoma	9.0	39	6.5	19	14.0	20	0.011*	9.2	33	8.1	6	0.772*
Head and neck	3.9	17	1.7	5	8.4	12	< 0.001*	4.7	17	0.0	0	0.091^
Malignant melanoma	4.8	21	4.5	13	5.6	8	0.607*	3.9	14	9.5	7	0.063^
Lung	3.2	14	2.4	7	4.9	7	0.138^	3.1	11	4.1	3	0.716^
Sarcoma	3.2	14	4.1	12	1.4	2	0.107^	3.1	11	4.1	3	0.716^
Leukemia	2.5	11	2.4	7	2.8	4	0.517^	2.5	9	2.7	2	1.000^
Bone marrow	2.3	10	2.1	6	2.8	4	0.430^	2.2	8	2.7	2	0.682^
Other cancer sites	13.8	60	10.7	31	20.3	29	0.006*	11.9	43	23.0	17	0.012*
**In active cancer treatment**							0.244*					0.672*
Yes	34.6	150	36.4	106	30.8	44		35.0	126	32.4	24	
No	65.4	284	63.6	185	69.2	99		65.0	234	67.6	50	
**Late and long-term effects**												
Fatigue	59.2	257	63.2	184	51.0	73	0.015*	71.4	257	–	–	
Sleep disorder	41.5	180	49.5	144	25.2	36	< 0.001*	50.0	180	–	–	
Hot flashes	39.2	170	49.8	145	17.5	25	< 0.001*	47.2	170	–	–	
Nerve damage (polyneuropathy)	38.0	165	45.7	133	22.4	32	< 0.001*	45.8	165	–	–	
Pain	36.6	159	43.3	126	23.1	33	< 0.001*	44.2	159	–	–	
Decreased muscle strength and mobility	36.4	158	42.6	124	23.8	34	< 0.001*	43.9	158	–	–	
Cognitive challenges	34.3	149	44.3	129	14.0	20	< 0.001*	41.4	149	–	–	
Sexual problems	31.6	137	28.9	84	37.1	53	0.084*	38.1	137	–	–	
Gained weight	27.2	118	33.3	97	14.7	21	< 0.001*	32.8	118	–	–	
Anxiety or depression	22.1	96	24.4	71	17.5	25	0.103*	26.7	96	–	–	
Early menopause	20.7	90	30.9	90	0.0	0	< 0.001*	25.0	90	–	–	
Mouth/tooth-problems and reduced taste	19.4	84	22.3	65	13.3	19	0.025*	23.3	84	–	–	
Urinary tract problems	18.0	78	15.1	44	23.8	34	0.027*	21.7	78	–	–	
Diarrhea	18.0	78	19.9	58	14.0	20	0.129*	21.7	78	–	–	
Constipation	16.6	72	18.6	54	12.6	18	0.116*	20.0	72	–	–	
Lymphedema	15.0	65	17.9	52	9.1	13	0.016*	18.1	65	–	–	
Heart- and lung problems	9.0	39	10.0	29	7.0	10	0.309*	10.8	39	–	–	
Reduced fertility	7.6	33	6.9	20	9.1	13	0.413*	9.2	33	–	–	
Weight loss	6.9	30	5.8	17	9.1	13	0.210*	8.3	30	–	–	
Other late and long-term effects	15.0	65	14.4	42	16.1	23	0.651*	18.1	65	–	–	

*Pearson chi-square test

^Fisher exact test

**Independent sample t-test

^1^Multiple choice

The most commonly reported cancer type was breast cancer (38.7%, *n* = 168) followed by gastrointestinal cancers (14.3%, *n* = 62), male genital cancer (11.1%, *n* = 48), female genital cancer (8.6%, *n* = 36), and lymphoma (9.0%, *n* = 39, Table 1).

### Late and long-term effects after cancer diagnosis and treatment

A high percentage of the participants (83%, *n* = 360, Fig. 1) reported to suffer from late and long-term effects of cancer diagnosis and treatment with a mean number of 5.1 late and long-term effects reported (SD 3.92, range 0-15). Most common was *fatigue* reported by 59.2% (*n* = 257) of the participants, followed by *sleep disorder* (41.5%, *n* = 180), *hot flashes* (39.2%, *n* = 170), *nerve damage* (polyneuropathy, 38.0%, *n* = 165), and *pain* (36.6%, *n* = 159, Table 1).

The participants reporting late and long-term effects were somewhat younger (58.6 years vs. 62.2 years, *p* = 0.011) and more likely to have college or university education (65.5% vs. 52.7%, *p* = 0.038, Table 1). No significant differences were found regarding gender (*p* = 0.127), income (*p* = 0.545), place of residence (*p* = 0.210), whether they lived alone or not (*p* = 0.437), or whether they were in active cancer treatment at the time of the survey (*p* = 0.672, Table 1). Although late and long-term effects were not associated with any specific cancer site (*p* > 0.05), participants suffering from cancers other than those mentioned above were less likely to experience late and long-term effects than those suffering from the listed cancer sites (*p* = 0.012).

### CAM used for late and long-term effects after cancer diagnosis and treatment

A total of 42.5% (*n* = 153) of the participants with late and long-term effects reported having used CAM to treat these complaints, more women (51.4%, *n* = 127) than men (23.0%, *n* = 26, *p* < 0.001, Table 2). The CAM users were in general older than the non-users (mean age of 56.9 compared to 49.8, *p* = 0.009), and more likely to have university education (*p* = 0.002, Table 2).

**Table 2:** Associations of for CAM use to treat late and long-term effects

	Total CAM use			CAM provider			Natural remedies			Self-help practices		
	% (***n*** = 153)	% (***n*** = 207)	***p***-value	% (***n*** = 80)	% (***n*** = 280)	***p***-value	% (***n*** = 47)	% (***n*** = 313)	***p***-value	% (***n*** = 95)	% (***n*** = 265)	***p***-value
**Sex**	Yes	No	< 0.001*	Yes	No	< 0.001*	Yes	No	0.109*	Yes	No	< 0.001*
Women	51.4 (127)	48.6 (120)		27.9 (69)	72.1 (178)		15.0 (37)	85.0 (210)		34.0 (84)	66.0 (163)	
Men	23.0 (26)	77.0 (87)		9.7 (11)	90.3 (102)		8.8 (10)	91.2 (103)		9.7 (11)	90.3 (102)	
**Age**			0.045*			0.084*			0.779*			0.035*
19-50 years	50.0 (42)	50.0 (42)		26.2 (22)	73.8 (62)		11.9 (10)	88.1 (74)		35.7 (30)	64.3 (54)	
51-64 yeras	44.7 (72)	55.3 (89)		24.8 (40)	75.2 (121)		12.4 (20)	87.6 (141)		26.7 (43)	73.3 (118)	
65-82 years	33.3 (38)	66.7 (76)		14.9 (17)	85.1 (97)		14.9 (17)	85.1 (97)		19.3 (22)	80.7 (92)	
Mean age, years (SD)	56.9 (10.71)	49.8 (10.36)	0.009**	56.1 (10.56)	59.3 (10.52)	0.019**	60.0 (11.40)	58.3 (10.47)	0.310**	55.5 (10.60)	59.6 (10.40)	0.001**
**Education**			< 0.001*			0.013^			0.360^			0.010*
Primary school	15.8 (3)	84.2 (16)		5.3 (1)	94.7 (18)		5.3 (1)	94.7 (18)		10.5 (2)	89.5 (17)	
Secondary school	32.4 (34)	67.6 (71)		15.2 (16)	84.8 (89)		10.5 (11)	89.5 (94)		18.1 (19)	81.9 (86)	
College/ University	48.9 (115)	51.1 (120)		26.4 (62)	73.6 (173)		14.9 (35)	85.1 (200)		31.5 (74)	68.5 (161)	
**Household income**			0.104*			0.226*			0.624^			0.269*
Low (Less than EUR 40,000)	31.4 (11)	68.6 (24)		14.3 (5)	85.7 (30)		8.6 (3)	91.4 (32)		14.3 (5)	85.7 (30)	
Middle EUR 40,000 to 79,000)	38.8 (50)	61.2 (79)		21.7 (28)	78.3 (101)		12.4 (16)	87.6 (113)		24.8 (32)	75.2 (97)	
High (EUR 80,000 or more)	48.8 (82)	51.2 (86)		25.6 (43)	74.4 (125)		15.5 (26)	84.0 (142)		29.8 (50)	70.2 (118)	
No reply	33.3 (9)	66.7 (18)		11.1 (3)	88.9 (24)		7.4 (2)	92.6 (25)		29.6 (8)	70.4 (19)	
**Household**^**1**^												
Live alone	43.4 (36)	56.6 (47)	0.854*	22.9 (19)	77.1 (64)	0.867*	9.6 (8)	90.4 (75)	0.292*	27.7 (23)	72.3 (60)	0.755*
Live with a partner	43.6 (113)	56.4 (146)	0.488*	22.8 (59)	77.2 (200)	0.684*	14.7 (38)	85.3 (221)	0.145*	27.0 (70)	73.0 (189)	0.660*
Live with own children	40.8 (29)	59.2 (42)	0.753*	19.7 (14)	80.3 (57)	0.571*	12.7 (9)	87.3 (62)	0.916*	25.4 (18)	74.6 (53)	0.825*
**Place of residence**			0.558*			0.957^			0.656^			0.170^
South-East	45.5 (85)	54.5 (102)		23.0 (43)	77.0 (144)		11.8 (22)	88.2 (165)		31.0 (58)	69.0 (129)	
South	53.3 (8)	46.7 (7)		26.7 (4)	73.3 (11)		20.0 (3)	80.0 (12)		33.3 (5)	66.7 (10_)	
West	36.0 (31)	64.0 (55)		20.9 (18)	79.1 (68)		11.6 (10)	88.4 (76)		17.4 (15)	82.6 (71)	
Central (Trøndelag)	40.0 (12)	60.0 (18)		23.3 (7)	76.7 (23)		16.7 (5)	83.3 (26)		23.3 (7)	76.7 (23)	
North	40.5 (17)	59.5 (25)		19.0 (8)	81.0 (34)		16.7 (7)	83.3 (35)		23.8 (10)	76.2 (32)	

*Pearson chi-square test

^Fisher exact test

**Independent sample t-test

^1^Multiple choice

Participants suffering from breast cancer (58.2%, *p* < 0.001) and female genital cancer (58.8%, *p* = 0.043) were more likely to use CAM while participants suffering from male genital cancer were less likely to use CAM (25.0%, *p* = 0.025). Participants with gastrointestinal cancers were also less likely to use CAM (25.0%, *p* = 0.008); however, not at a significant level when adjusted for gender (*p* = 0.051, Table 3).

**Table 3:** Cancer-related associations for use of CAM to treat late and long-term effects from cancer diagnosis and treatment

	Total CAM use			CAM provider			Natural remedies			Self-help practices		
	% (***n*** = 153)	% (***n*** = 207)	***p***-value	% (***n*** = 80)	% (***n*** = 207)	***p***-value	% (***n*** = 47)	% (***n*** = 313)	***p***-value	% (***n*** = 95)	% (***n*** = 265)	***p***-value
**Cancer site**^**1**^	Yes	No		Yes	No		Yes	No		Yes	No	
Breast	58.2 (85)	41.8 (61)	< 0.001*	35.6 (52)	64.4 (94)	< 0.001*	13.7 (20)	86.3 (126)	0.765*	39.0 (57)	61.0 (89)	< 0.001*
Gastro intestinal	25.0 (12)	75.5 (36)	0.008*	8.3 (4)	91.7 (44)	0.013*	14.6 (7)	85.4 (41)	0.736*	18.8 (9)	81.3 (39)	0.197*
Male genitalia	25.0 (9)	75.0 (27)	0.025*	8.3 (3)	91.7 (33)	0.035*	8.3 (3)	91.7 (33)	0.600^	11.1 (4)	88.9 (32)	0.028*
Female genitalia	58.8 (20)	41.2 (14)	0.043*	29.4 (10)	70.6 (24)	0.289*	20.6 (7)	79.4 (27)	0.181^	44.1 (15)	55,9 (19)	0.014*
Lymphoma	27.3 (9)	72.7 (24)	0.063*	12.1 (4)	87.9 (29)	0.143*	6.1 (2)	93.9 (31)	0.284^	15.2 (5)	84,8 (28)	0.124*
Head and neck	52.9 (9)	47.1 (8)	0.372*	17.6 (3)	82.4 (14)	0.773^	17.6 (3)	82.4 (14)	0.474^	29.4 (5)	70,6 (12)	0.780^
Malignant melanoma	28.6 (4)	71.4 (10)	0.282*	21.4 (3)	78.6 (11)	1.000^	0.0 (0)	100 (14)	0.231^	14.3 (2)	85,7 (12)	0.371^
Lung	36.4 (4)	63.6 (7)	0.765^	18.2 (2)	81.8 (9)	1.000^	27.3 (3)	72.7 (8)	0.162^	9.1 (1)	90,9 (10)	0.300^
Sarcoma	63.6 (7)	36.4 (4)	0.215^	36.4 (4)	63.6 (7)	0.271^	27.3 (3)	72.7 (8)	0.162^	36.4 (4)	63,6 (7)	0.490^
Leukemia	0.0 (0)	100 (9)	0.012^	0.0 (0)	100 (9)	0.216^	0.0 (0)	100 (9)	0.612^	0.0 (0)	100 (9)	0.119^
Bone marrow	25.0 (2)	75.0 (6)	0.475^	25.0 (2)	75.0 (6)	1.000^	12.5 (1)	87.5 (7)	1.000^	0.0 (0)	100 (8)	0.116^
Other cancer sites	32.6 (14)	67.4 (29)	0.160*	11.6 (4)	88.4 (38)	0.075*	14.0 (6)	86.0 (37)	0.852*	20.9 (9)	79.1 (34)	0.387*
**In active cancer treatment**			0.427*			0.425*			0.144*			0.039*
Yes	39.7 (50)	60.3 (7)		24.6 (31)	75.4 (95)		9.5 (12)	90.5 (114)		19.8 (25)	80.2 (101)	
No	44.0 (103)	56.0 (131)		20.9 (49)	79.1 (185)		15.0 (35)	85.0 (199)		29.9 (70)	70.1 (164)	
**Late and long-term effects**												
Fatigue	47.5 (122)	52.5 (135)	0.003*	24.1 (62)	75.9 (195)	0.170*	13.6 (35)	86.4 (222)	0.616*	31.1 (80)	68.9 (177)	0.001*
Sleep disorder	53.3 (96)	46.7 (84)	< 0.001*	28.9 (52)	71.1 (128)	0.002*	15.6 (28)	84.4 (152)	0.159*	32.8 (59)	67.2 (121)	0.006*
Hot flashes	55.3 (94)	44.7 (76)	< 0.001*	30.0 (51)	70.0 (119)	< 0.001*	17.1 (29)	82.9 (141)	0.033*	35.9 (61)	64.1 (109)	< 0.001*
Nerve damage (polyneuropathy)	52.7 (87)	47.3 (78)	< 0.001*	26.7 (44)	73.3 (121)	0.062*	14.5 (24)	85.8 (141)	0.440*	35.8 (59)	64.2 (106)	< 0.001*
Pain	54.1 (86)	45.9 (73)	< 0.001*	30.2 (48)	69.8 (111)	0.001*	15.1 (24)	84.9 (135)	0.307*	35.8 (57)	64.2 (102)	< 0.001*
Decreased muscle strength and mobility	52.5 (83)	47.5 (75)	< 0.001*	30.4 (48)	69.6 (110)	< 0.001*	15.8 (25)	84.2 (133)	0.168*	32.9 (52)	67.1 (106)	0.013*
Cognitive challenges	63.1 (94)	36.9 (55)	< 0.001*	34.2 (51)	65.8 (98)	< 0.001*	17.4 (26)	82.6 (123)	0.038*	14.6 (62)	58.4 (87)	< 0.001*
Sexual problems	46.7 (64)	53.5 (73)	0.205*	24.1 (33)	75.9 (104)	0.505*	15.3 (21)	84.7 (116)	0.316*	29.2 (40)	70.8 (97)	0.343*
Gained weight	53.4 (63)	46.6 (55)	0.004*	32.2 (38)	67.8 (80)	0.001*	16.9 (20)	83.1 (98)	0.126*	30.5 (36)	69.5 (82)	0.216*
Anxiety or depression	55.2 (53)	44.8 (43)	0.003*	30.2 (29)	69.8 (67)	0.028*	15.6 (15)	84.4 (81)	0.383*	36.5 (35)	63.5 (61)	0.009*
Early menopause	66.7 (60)	33.3 (30)	< 0.001*	41.1 (37)	58.9 (53)	< 0.001*	16.7 (15)	83.3 (75)	0.240*	48.9 (44)	51.1 (46)	< 0.001*
Mouth/tooth-problems and reduced taste	56.0 (47)	44.0 (37)	0.004*	31.0 (26)	69.0 (58)	0.028*	14.3 (12)	85.7 (72)	0.702*	28.6 (24)	71.4 (60)	0.604*
Urinary tract problems	42.3 (33)	57.7 (45)	0.969*	20.5 (16)	79.5 (62)	0.682*	14.1 (11)	85.9 (67)	0.756*	26.9 (21)	73.1 (57)	0.904*
Diarrhea	52.6 (41)	47.4 (37)	0.042*	26.9 (21)	73.1 (57)	0.259*	16.7 (13)	83.3 (65)	0.285*	33.3 (26)	66.7 (52)	0.116*
Constipation	52.8 (38)	47.2 (34)	0.049*	31.9 (23)	68.1 (49)	0.027*	18.1 (13)	81.9 (59)	0.159*	33.3 (24)	66.7 (48)	0.135*
Lymphedema	55.4 (36)	44.6 (29)	0.020*	32.3 (21)	67.7 (44)	0.031*	15.4 (10)	84.6 (55)	0.538*	29.2 (19)	70.8 (46)	0.566*
Heart- and lung problems	46.2(18)	53.8 (21)	0.625*	25.6 (10)	74.4 (29)	0.587*	20.5 (8)	79.5 (31)	0.143*	28.2 (11)	71.8 (28)	0.785*
Reduced fertility	57.6 (19)	42.4 (14)	0.066*	27.3 (9)	72.7 (24)	0.464*	9.1 (3)	90.9 (30)	0.597^	36.4 (12)	63.6 (21)	0.173*
Weight loss	40.0 (12)	60.0 (18)	0.772*	16.7 (5)	83.3 (25)	0.445*	6.7 (2)	93.3 (28)	0.399^	23.3 (7)	76.7 (23)	0.692*
Other late and long-term effects	35.4 (23)	64.6 (42)	0.200*	15.4 (10)	84.6 (55)	0.143*	16.9 (11)	83.1 (54)	0.307*	21.5 (14)	78.5 (51)	0.327*

*Pearson chi-square test

^Fisher exact test

^1^As some participants might have been diagnosed with more than one cancer form the total number of cancer sites extend the number of participants in the study

The modalities most used were self-help practices (26.4%, *n* = 95) like relaxation therapy (19.2%, *n* = 69), yoga (14.2%, *n* = 51) or meditation/mindfulness (12.8%, *n* = 46, Table 4) and most users of self-help practices (86.3%) reported perceived improvements with these practices. Consultations with CAM providers were also common, reported by 22.2% (*n* = 80), with 90.0% reporting improvements after seeing a CAM provider. Most reported were massage (12.5%, *n* = 45), acupuncture (8.9%, *n* = 32), and osteopathy (4.2%, *n* = 15). None had used Rosen therapy and only one had used coaching to treat late and long-term effects. Few (13.1%, *n* = 47) had used natural remedies, mostly Omega 3 fatty acids (6.4%, *n* = 23), ginger (3.9%. *n* = 14), turmeric/curcumin (3.6%, *n* = 13), and blueberry/blueberry extract (2.2%, *n* = 8, Table 4), of whom 46.0% experienced improvements of their symptoms.

**Table 4:** CAM modalities used to treat late and long-term effects of cancer diagnosis and treatment

	Total	Late and long-term effect(s) (multiple choice)					Adverse effects of CAM treatment
		Fatigue	Sleep disorder	Hot flashes	Nerve damage (polyneuropathy)	Pain	
	% (***n*** = 360)	% (***n*** = 257)	% (***n*** = 180)	% (***n*** = 170)	% (***n*** = 165)	% (***n*** = 159)	% (***n*** = 16)
**CAM provider**	**22.2 (80)**	**24.1 (62)**	**28.9 (52)**	**30.0 (51)**	**26.7 (44)**	**30.2 (48)**	**8.8 (7)**
Massage/ aromatherapy	12.5 (45)	13.6 (35)	15.5 (28)	15.9 (27)	16.4 (27)	18.9 (30)	6.7 (3)
Acupuncture	8.9 (32)	8.6 (22)	13.3 (24)	13.5 (23)	10.9 (18)	10.7 (17)	9.4 (3)
Osteopathy	4.2 (15)	3.5 (9)	4.4 (8)	6.5 (11)	5.5 (9)	6.3 (10)	0.0 (0)
Naprapathy	2.5 (9)	3.1 (8)	4.4 (8)	3.5 (6)	1.2 (2)	3.1 (5)	22.2 (2)
Reflexology	1.4 (5)	1.6 (4)	1.7 (3)	2.9 (5)	1.8 (3)	1.9 (3)	0.0 (0)
Healing	1.1 (4)	1.6 (4)	2.2 (4)	1.2 (2)	1.8 (3)	2.5 (4)	0.0 (0)
Homeopathy	1.1 (4)	1.6 (4)	1.7 (3)	1.2 (2)	1.2 (2)	0.6 (1)	0.0 (0)
Herbal therapy	0.6 (2)	0.8 (2)	0.0 (0)	1.2 (2)	1.2 (2)	0.6 (1)	0.0 (0)
**Natural remedies**	**13.1 (47)**	**13.6 (35)**	**15.6 (28)**	**17.1 (29)**	**14.5 (24)**	**15.1 (24)**	**6.4 (3)**
Omega 3 fatty acids	6.4 (23)	6.6 (17)	8.3 (15)	8.2 (14)	4.2 (7)	7.5 (12)	4.3 (1)
Ginger	3.9 (14)	3.5 (9)	2.8 (5)	5.3 (9)	4.2 (7)	3.8 (6)	7.1 (1)
Turmeric / curcumin	3.6 (13)	4.3 (11)	4.4 (8)	5.9 (10)	6.7 (11)	3.1 (5)	7.7 (1)
Green tea	1.9 (7)	2.3 (6)	1.7 (3)	2.9 (5)	3.0 (5)	0.6 (1)	0.0 (0)
Blueberries / blueberry extract	2.2 (8)	1.9 (5)	2.2 (4)	2.4 (4)	1.2 (2)	1.9 (3)	1.3 (1)
Garlic	1.7 (6)	1.9 (5)	2.2 (4)	2.4 (4)	1.8 (3)	0.6 (1)	0.0 (0)
*Aloe Vera*	1.7 (6)	0.8 (2)	1.1 (2)	1.8 (3)	1.8 (3)	1.9 (3)	0.0 (0)
Q10	0.8 (3)	1.2 (3)	1.1 (2)	1.2 (2)	1.2 (2)	1.9 (3)	0.0 (0)
Chaga	0.6 (2)	0.8 (2)	1.1 (2)	1.2 (2)	0.6 (1)	0.6 (1)	0.0 (0)
**Self-help practices**	**26.4 (95)**	**31.1 (80)**	**32.8 (59)**	**35.9 (61)**	**35.8 (59)**	**35.8 (57)**	**7.4 (7)**
Relaxation	19.2 (69)	22.2 (57)	21.7 (39)	24.7 (42)	23.6 (39)	25.2 (40)	4.3 (3)
Yoga	14.2 (51)	16.7 (43)	18.9 (34)	21.8 (37)	18.8 (31)	23.9 (38)	9.8 (5)
Meditation/Mindfulness	12.8 (80)	15.6 (40)	18.3 (33)	20.6 (35)	16.4 (27)	20.1 (32)	6.3 (5)
Visualization	1.7 (6)	1.6 (4)	1.7 (3)	2.9 (5)	2.4 (4)	1.9 (3)	0.0 (0)
Tai chi / chi gong	1.7 (6)	2.3 (6)	2.8 (5)	2.9 (5)	3.0 (5)	2.5 (4)	1.7 (1)
Music therapy	1.1 (4)	1.2 (3)	1.1 (2)	0.6 (1)	1.2 (2)	1.3 (2)	0.0 (0)
Art Therapy	0.6 (2)	0.8 (2)	1.1 (2)	1.2 (2)	1.2 (2)	1.3 (2)	0.0 (0)
**Total CAM use**	**42.5 (153)**	**47.5 (122)**	**53.3 (96)**	**55.3 (94)**	**52.7 (87)**	**54.1 (86)**	**10.5 (16)**

Participants suffering from *early menopause*, *cognitive challenges*, and *reduced fertility* were; however, the most frequent users of CAM in general (66.7, 63.1, and 57.6% respectively, Table 3). Participants suffering from *fatigue, sleep disorders, hot flashes, nerve damage (polyneuropathy),* and *pain (n = 159)* were most likely to use *relaxation therapy* (22-25%), *yoga* (17-24%), and *meditation/mindfulness* (16-20%, Table 4) to alleviate their late and long-term effects.

Few (10.5%, *n* = 16) experienced adverse effects of the CAM treatment, and only mild and moderate adverse effects were reported, mainly from yoga (*n* = 5), meditation/mindfulness (*n* = 5), massage (*n* = 3), acupuncture (*n* = 3), and relaxation techniques (*n* = 3, Table 4). Details of adverse effects were not collected, but all were described as mild or moderate.

## Discussion

### Main findings

In the present study three out of four participants reported experiencing late and long-term effects from cancer and its treatment, mostly *fatigue*, *sleep disturbances*, *hot flashes*, *nerve damage* (polyneuropathy), and *pain.* Participants reported a mean number of 5.1 different late and long-term effects, and younger women with higher income and education were more frequently affected. A total of 42.5% of the participants with late and long-term effects reported having used CAM to treat this complaint. Most used were self-help practices (26.4%) such as relaxation therapy (19.2%), yoga (14.2%) and meditation (12.8%). A high percentage of CAM users reported self-perceived improvements of their symptoms (86.3% after use of self-help practices, 90.0% after visits to CAM providers), and few experienced adverse effects of the CAM treatment.

### Adverse effects of CAM treatments

Of the CAM users in the present study 10.5% experienced adverse effects of a CAM treatment. These reported adverse effects were only mild to moderate and related to yoga, meditation, massage, acupuncture, and relaxation techniques. This is in line with general findings that many CAM treatments are associated with mild and transient adverse events only and that serious CAM-related adverse events are rare when used appropriately [81].

Adverse effects reported in the literature after yoga mainly concern the musculoskeletal system [82] while anxiety, traumatic re-experiencing, and emotional sensitivity are most commonly reported after meditation [83]. The most common adverse effects reported from massage are increased discomfort/soreness, bruising, headache, and tiredness/fatigue [84]. For acupuncture are local pain, bruising, minor bleeding, and orthostatic problems the most commonly reported adverse effects [85]. People report occasionally anxiety, intrusive thoughts, or fear of losing control due to relaxation techniques [86]. CAM treatments can potentially interact with other treatments, which is an important safety issue for patients receiving concomitant treatment [81].

### Agreements and disagreements with other studies

The findings of 83% reporting late and long-term effects from cancer diagnosis and treatment is somewhat higher than what was found in an earlier Norwegian study where 61.5% of the participants reported at least one late effect [87]. The discrepancy might be due to the fact that the latter study was conducted among adolescent and young adult cancer survivors who thanks to their younger age recovered more easily from these effects. This is suspected as the latter study also reported a lower number of different late and long-term effects experienced by each participant (2.4 vs. 5.1 respectively).

We were unable to identify other studies describing CAM use specifically for late and long-term effects of cancer and cancer treatment. A recent systematic review assessing the existing instruments for identifying, diagnosing, and managing late effects of cancer survivors, found that none of the existing studies adequately addressed this, pointing to a lack of suitable research studies [88]. We will therefore compare our findings to the use of CAM for adverse effects of cancer diagnosis and treatment as adverse effects also can persist long term. We will also discuss our findings with other studies reporting CAM used for the most frequently reported late and long-term effects reported in this study, namely *fatigue*, *sleep disorder*, *early menopause (hot flashes), nerve damage*, and *pain*.

A cross-sectional survey of individuals who currently have or previously had cancer in Norway assessing all-time use of CAM for cancer-related complaints, reported that 79% of the respondents (*n* = 346) had used some form of CAM; 33% (*n* = 143) had seen a CAM provider, 52% (*n* = 230) had used natural remedies, while 58% (*n* = 253) had used self-help practices. Most of the cancer patients used CAM to increase the quality of life, for coping with the cancer disease, or for relaxation/well-being (64-94% )[79]. Overall, this use is noticeably higher than CAM use for late and long-term effects alone, particular use of natural remedies (52% vs 13%), and self-help practices (58% vs 26%). The reason for this higher usage may be that CAM use was reported for a wide variety of reasons including, but not limited to, late and long-term effects.

As in the present study, Eustachi et al. found a rather high percentage of participants (37%) in Germany, diagnosed with cancer 1-20 years prior to the study, using CAM [89]. This was also the case among Malaysian cancer patients where 60.8% reported using CAM for management of chemotherapy-related adverse effects [90]. A larger variation in CAM use for adverse effects of cancer diagnosis and treatment ranging from 1 to 70% was; however, found in a recent systematic review examining CAM use in cancer patients on a more general level [91]. The reason for this generally high use of CAM for adverse effects and late and long-term effects from cancer diagnosis and treatment might be twofold: Firstly, conventional cancer care might lack the levels of healthcare staff and infrastructure to address the needs of cancer patients suffering from late and long-term effects [3] and secondly, treatment options for the late and long-term effects experienced are limited within conventional health care [33], or associated with severe adverse effects [72].

Our findings of frequent visits to CAM providers among women experiencing hot flashes and early menopause due to cancer treatment are in line with an Australian study reporting CAM use by women with invasive breast cancer. It found that women visiting CAM providers had higher Menopause Quality of Life Questionnaire scores on average 92 weeks after being diagnosed with cancer than the women who did not [92]. The women in the present study did; however, visit CAM providers somewhat more frequently for these symptoms (40% for early menopause and 30% for hot flashes versus 10.6% in the Australian study). One of the reasons for this discrepancy might be that the Australian study limited the use to the last 12 months and that the participants were only 1-2 years post diagnosis, while the highest use of CAM among female cancer patients in Norway was found to be 1-5 years after being diagnosed with cancer [93].

In the present study, 44% of the participants reported cancer-related pain and 54% thereof used CAM to manage it. In accordance with this, Jaradat et al. [94], Abuelgasim et al. [95], and Al-Naggar et al. [96] found that their patients treated cancer-related pain with CAM; however, to a somewhat lesser degree (1.4-20%). The discrepancy for this is likely to be different definitions of CAM and different timeframes of CAM use. In a study of CAM for management of pain in general, Rosenberg et al. [97] found that 52% of the participants had used CAM for relief of chronic pain, and in accordance with our findings, massage was among the most frequently used therapies.

### CAM use and clinical guidelines

A range of CAM modalities for supportive care of cancer have been evaluated for inclusion in clinical guidelines such as guidelines by the European Society for Medical Oncology (ESMO )[28], the German Guideline Program in Oncology (GGPO )[98], and the American Society of Clinical Oncology (ASCO) [99] endorsed guidelines by the Society of Integrative Oncology. These refer to prevention and treatment of adverse effects in general, whether acute or long-term. We have therefore discussed the most commonly used modalities for the five most reported symptoms in the context of these guidelines. These guidelines include a risk-benefit evaluation and unless otherwise stated, the mentioned treatments are associated with minor and transient adverse effects only.

#### Cancer-related fatigue (CRF)

In the present study fatigue was the most frequently reported late and long-term effect, reported by 59% of the participants of whom 43% had used CAM. Relaxation therapy (18%), yoga (14%), meditation/mindfulness (13%), and massage/aromatherapy (12.5%) were most frequently used to treat CRF. Clinical guidelines recommend the following modalities: yoga (ESMO, GGPO, ASCO), mindfulness (ESMO, ASCO; GGPO: could be considered), acupuncture (ASCO; ESMO, GGPO: could be considered), tai chi/qigong (GGPO; ASCO: could be considered), ginseng (ESMO, GGPO: could be considered). This is in line with the peer-reviewed and systematic evidence summaries published on NAFKAM’s website CAM Cancer [100], which reports beneficial results for mindfulness, yoga, tai chi, ginseng, music therapy, and promising yet not fully conclusive results for acupuncture and massage. The number of cancer survivors reporting CRF was somewhat higher in this study compared to earlier Norwegian studies showing that 25-35% of long-term survivors of breast cancer [24, 101], lymphoma [102], and cervical [103] cancer are affected by CRF [10].

#### Sleep disturbances

In the present study 42% of the participants reported sleep disturbance of whom 53% used CAM to treat this. This is somewhat higher than what Pearson et al. found in a general population suffering from insomnia or trouble sleeping where 4.5% of non-institutionalized adults aged 18 and above reported using CAM to treat their sleep problem during the past year [104]. The discrepancies might be due to a younger population, with fewer comorbidities and a shorter time frame of use in the latter study. The present study revealed that relaxation therapy (22%), yoga (19%), meditation/mindfulness (18%), massage (14%), and acupuncture (13%) were the most used approaches. Treatment guidelines recommend that tai chi/qigong (GGPO) may be used, and that (gentle) yoga, mindfulness/meditation, and acupuncture could be considered (GGPO, ASCO). CAM Cancer’s evidence summaries have found moderate evidence that yoga improves sleep quality in cancer patients and some positive but not fully conclusive evidence that mindfulness improves sleep [100]. Systematic reviews have further reported improvements in sleep quality in patients with insomnia from acupuncture [105, 106], meditation [106], and massage [107] but not relaxation therapy [107]. The number of cancer survivors reporting sleep disturbances were found to be in accordance with earlier studies finding that 31- 51% report sleep disturbance during the cancer survivorship period [108, 109].

#### Hot flashes

In the present study 50% of the women reported hot flashes, 62% when limited to those with female genital cancer and breast cancer. Of the women suffering from hot flashes, 59% had used CAM to treat their complaints. Women suffering from hot flashes were most likely to use relaxation therapy (22%), yoga (19%), meditation/mindfulness (18%), massage/aromatherapy (16%), and acupuncture (14%).

Treatment guidelines state that for hot flashes acupuncture can (ASCO, GGPO) be considered. According to GGPO, yoga, meditation/mindfulness, and black cohosh (*Actaea racemosa*) could be considered. A Cochrane review has further reported relaxation techniques to be both safe and beneficial [33]. CAM Cancer’s evidence summary of acupuncture [110] reports best evidence for reducing the severity of hot flashes.

The number of women reporting hot flashes in the present study were in accordance with what was found in earlier studies suggesting that 59-65% of breast cancer survivors experience hot flashes post treatment [111, 112].

#### Chemotherapy-induced peripheral neuropathy (CIPN)

In the present study 38% of the participants reported to suffer from CIPN, of whom 53% reported using CAM to treat the complaint. Most used were relaxation therapy (24%), yoga (19%), massage (16%), meditation (16%), and acupuncture (11%). A total of 14.5% reported using herbs, with curcumin/turmeric (7%) most frequently used.

Clinical guidelines do not recommend any CAM approaches in the prevention of CIPN (ESMO, ASCO, GGPO). For the treatment of CIPN, the ESMO guidelines state that acupuncture might be considered. Several CAM modalities have been investigated for the prevention and treatment of CIPN [39], reporting mixed results for natural products and dietary supplements as well as preliminary encouraging findings for acupuncture, massage, and mind-body therapy [39]. The number of participants reporting CIPN in the present study were in accordance with earlier studies finding that 30-50% of cancer patients treated with chemotherapy experienced CIPN 6 months or later post treatment dependent of chemotherapy used [36, 37].

#### Cancer-related pain

In the present study 37% of the participants reported to suffer from cancer-related pain, of whom 54% used CAM to ease the pain. Mostly used were relaxation therapy (25%), yoga (24%), meditation/mindfulness (20%), massage (19%), and acupuncture (11%) for pain management.

GGPO recommends that acupuncture should be considered for joint pain in breast cancer patients, as well as in general oncological populations for tumor pain. It considers the evidence for massage to be insufficient. The ASCO guidelines [113] give a weak recommendation for massage, acupuncture, and music therapy based on low quality evidence and that the benefits outweigh the harms. Moderate recommendations are given for mindfulness, relaxation, and guided imagery based on intermediate quality of evidence.

CAM Cancer’s evidence summary on acupuncture for cancer pain [114] reports positive results from newer systematic review for adding acupuncture to conventional treatment. Very low-quality evidence supports the use of massage for relief of short-term pain. Evidence on longer term pain is too heterogeneous for firm conclusions due to variations in types of massage studied and comparators against which these are assessed. For yoga, the evidence is not conclusive and for Mindfulness Based Stress Reduction (MBSR) no convincing effects on pain have been reported. The number of participants reporting cancer related pain in the present study were in accordance with earlier studies finding that 33-40% of cancer survivors suffer from chronic pain after their curative treatment was completed [115, 116].

### Strengths and limitations

The main strengths of the study are the high response rate, the adequate study power, an age distribution similar to adult cancer survivors in Norway, in addition to the wide range of late and long-term effects and cancer modalities studied. The study must; however, be understood in the light of some limitations.

The main limitations of the study is that the participants do not fully represent the total cancer population in Norway with more female participants than female cancer patients in general (67% vs 46%), and that the cancer diagnosis was self-reported. Further, the study population was more likely to have a university degree [117]. This might have led to an over-representation of total CAM users as female gender, as well as higher education are positively associated with CAM use. This applies also for the high number of late and long-term effects reported in this study as late and long-term effects were associated with college/university education.

Other limitations are the self-reported late and long-term effects, leading to possible bias concerning how to understand late and long-term effects, and the lack of information of which individual conventional cancer treatments the participants have received. The high number of participants reporting late and long-term effects may be due to difficulties in distinguishing late and long-term effects from adverse effects of cancer diagnosis and treatment and complaints caused by other reasons as several of these complaints are common also in the general population.

The question regarding reason for CAM use covered both adverse effects and late and long-term effects of cancer diagnosis and treatment. The number of participants reporting using CAM for late and long-term effects reported in this study might therefore be somewhat overestimated although all reported having late and long-term effects from their cancer diagnosis and treatment. As many reported more than one late and long-term effect, we cannot say for certain that the therapy was used for each particular late and long-term effect, we can only be certain that participants suffering from the specific late and long-term effect used the reported therapies to treat a late or long-term effect/adverse effect of the cancer diagnosis and treatment.

We cannot completely rule out the possibility that people with a special interest in CAM were more likely to be among the responders as the invitation to participate mentioned that CAM use was one of the topics together with diet and use of dietary supplements. No emphasis was put on late and long-term effects in the invitation, so we have no reason to believe that people suffering late and long-term effects of cancer diagnosis and treatment are overrepresented in the sample.

### Implication of the findings

The high number of cancer survivors experiencing late and long-term effects of their cancer diagnosis and treatment indicate that there is a need for a more comprehensive follow-up protocol with focus on more treatment options for late and long-term effects. In recent years, the attention around late and long-term effects of cancer diagnosis and treatment *has* increased in Norway and possible causes and treatment options have been explored [3]. National [68] as well as regional [69] competence centers are under establishment with the aim to improve patient information and guidance on how to live with and manage late and long-term effects of cancer diagnosis and treatment [68, 69]. Although the Norwegian guidelines for treating late and long-term effects of cancer diagnosis and treatment do not recommend CAM therapies, it might contribute to the management of late and long-term effects.

CAM modalities are to some degree incorporated into symptom management strategies among people suffering from late and long-term effects from cancer treatment [27]. Although the overall evidence is generally inconclusive for complete resolution of the late and long-term effects, several studies have demonstrated that the use of CAM can reduce these symptoms with few or no adverse effects [27].

To our knowledge this is the first study that systematically maps different CAM modalities used for a wide variation of late and long-term effects of cancer diagnosis and treatment. Since late and long-term effects greatly influence cancer survivors’ quality of life and have huge socioeconomic consequences on individual and societal levels, approaches to support people recover from cancer diagnosis and treatment are essential. With insights gained from this study, national and regional competence centers as well as future action programs on late and long-term effects can guide patients towards modalities with high satisfaction and low risk for their specific complaint. This may contribute to reduce suffering and improve quality of life for those struggling with late and long-term effects.

## Conclusion

A large proportion of cancer patients in Norway suffer from a wide range of late and long-term effects of cancer diagnosis and treatment, and they use CAM to treat these complaints to a rather high degree. Relaxation therapy, yoga, meditation, massage, and acupuncture were the most frequently used therapies regardless of complaint. The therapies used have generally shown to be both safe and beneficial for the respective complaints, indicating that the participants seem to be well informed about the choices they make.

## Data Availability

The dataset this paper has been based on has not been deposited in any repository. All dataset and materials are available from the corresponding author upon reasonable request. Applicants for any data must; however, be prepared to conform to Norwegian privacy regulations.

## Abbreviations

CAM: Complementary and Alternative Medicine
NCS: Norwegian Cancer Society
I-CAM-Q: International Questionnaire to Measure Use of Complementary and Alternative Medicine
NAFKAM: National Research Center in Complementary and Alternative Medicine
NSD: Norwegian Centre for Research Data
MBSR: Mindfulness Based Stress Reduction
CRF: Cancer-related fatigue
CIPN: Chemotherapy-induced peripheral neuropathy
